# A man with a long history of pruritus

**DOI:** 10.1002/jha2.79

**Published:** 2020-08-18

**Authors:** Katherin Blanco, Andrea Cristiani, Maria Eugenia Mazzei, Cecilia Guillermo, Gimena dos Santos

**Affiliations:** ^1^ Hematology Department Hospital de Clinicas Doctor Manuel Quintela Montevideo Uruguay; ^2^ Pathology Department Hospital de Clinicas Doctor Manuel Quintela Montevideo Uruguay; ^3^ Dermatology Department Hospital de Clinicas Doctor Manuel Quintela Montevideo Uruguay

**FIGURE 1:**
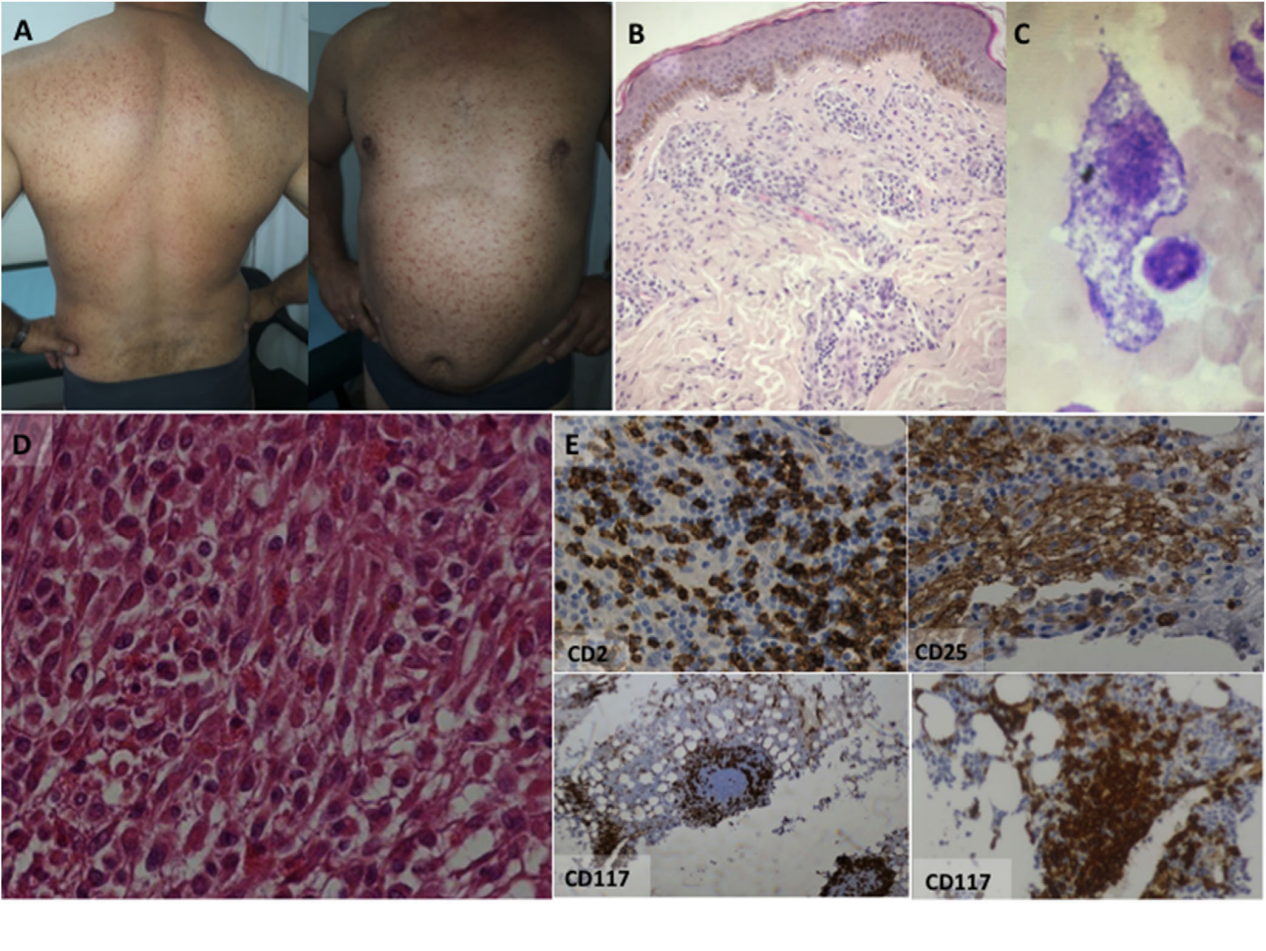
Systemic mastocytosis. (A) Hyperpigmented macules and papules on the trunk and extremities. (B) Dermis is infiltrated by aggregates of spindle‐shaped mast cells that are also present on bone marrow aspirates (C) and biopsy (D). A characteristic pattern of CD2, CD25, and CD117 displays positivity by immunohistochemical staining. B and D, H&E; C, May Grumwald‐Giemsa

A 49‐year‐old male with no past medical history was referred to the hematology clinic by his dermatologist with the results of a skin biopsy. The patient had a long history of intense pruritus and small tan to brown macules and papules distributed on the trunk and extremities (Figure 1, image A). Areas exposed to light were spared. The skin biopsy was positive for urticaria pigmentosa as it showed the presence of mast cells (MC) infiltrating the upper and lower dermis with increased melanization of the basal layer of the epidermis (Figure 1, image B). His laboratory tests included normal cell blood counts, biochemistry, and lactate dehydrogenase, and an increase in his baseline serum tryptase levels (46.6 ng/mL; normal range: 5 ‐15 ng/mL). Bone marrow aspirates revealed the presence of 7% of spindle‐shaped MC (Figure 1, image C), with an aberrant immunophenotype with coexpression of CD2, CD25, and CD117 by flow cytometry. Histologic examination of the bone marrow was remarkable for numerous foci of morphologically abnormal MC infiltrating perivascular and paratrabecular spaces (Figure 1, image D), intense positivity for CD2, CD25, and CD117 (Figure 1, image E), collagen deposition, and mild signs of dysplasia. *KIT* D816V mutation was found in the molecular assessment of the bone marrow by RT‐PCR.

Having met the major criterion (multifocal clusters of MC in at least one organ) and all three minor criteria (*KIT* point mutation at codon 816, aberrant expression of CD2 and CD25 in MC and baseline serum tryptase level > 20 ng/mL), the diagnosis of systemic mastocytosis (SM) according to WHO 2016 classification was established. The patient was diagnosed with indolent SM as no organ dysfunction was documented and computerized tomography scans were only positive for mild hepatomegaly and osteoblastic lesions on the skeletal survey, without osteolytic lesions or pathologic fractures. He is currently being managed with symptom control with antihistamines.

